# Immediate Bystander Cardiopulmonary Resuscitation to Sudden Cardiac Arrest During Sports is Associated with Improved Survival—a Video Analysis

**DOI:** 10.1186/s40798-021-00346-2

**Published:** 2021-07-22

**Authors:** Nicole M. Panhuyzen-Goedkoop, Hein J. Wellens, André L. M. Verbeek, Jan J. Piek, Ron J. G. Peters

**Affiliations:** 1 Heart Centre, Amsterdam University Medical Centre, AMC, Meibergdreef 9, 1105 AZ Amsterdam, the Netherlands; 2 Sports Medical Centre Papendal, Arnhem, the Netherlands; 3 Cardiac Research Centre, Maastricht, the Netherlands; 4 Department for Health Evidence, Radboud University Medical Centre, Nijmegen, the Netherlands

**Keywords:** Sudden cardiac death, Sudden cardiac arrest, Athlete, Sports participant, Sport, Soccer, Chest compressions, AED, Cardiopulmonary resuscitation

## Abstract

**Background:**

Sudden cardiac arrest (SCA) during sports can be the first symptom of yet undetected cardiovascular conditions. Immediate chest compressions and early defibrillation offer SCA victims the best chance of survival, which requires prompt bystander cardiopulmonary resuscitation (CPR).

**Aims:**

To determine the effect of rapid bystander CPR to SCA during sports by searching for and analyzing videos of these SCA/SCD events from the internet.

**Methods:**

We searched images.google.com, video.google.com, and YouTube.com, and included any camera-witnessed non-traumatic SCA during sports. The rapidity of starting bystander chest compressions and defibrillation was classified as < 3, 3–5, or > 5 min.

**Results:**

We identified and included 29 victims of average age 27.6 ± 8.5 years. Twenty-eight were males, 23 performed at an elite level, and 18 participated in soccer. Bystander CPR < 3 min (7/29) or 3–5 min (1/29) and defibrillation < 3 min was associated with 100% survival. Not performing chest compressions and defibrillation was associated with death (14/29), and > 5 min delay of intervention with worse outcome (death 4/29, severe neurologic dysfunction 1/29).

**Conclusions:**

Analysis of internet videos showed that immediate bystander CPR to non-traumatic SCA during sports was associated with improved survival. This suggests that immediate chest compressions and early defibrillation are crucially important in SCA during sport, as they are in other settings. Optimal use of both will most likely result in survival. Most videos showing recent events did not show an improvement in the proportion of athletes who received early resuscitation, suggesting that the problem of cardiac arrest during sports activity is poorly recognized.

## Key Points

- Analysis of internet videos showed that immediate bystander CPR to non-traumatic SCA during sports was associated with improved survival.

- Immediate chest compressions and early defibrillation are crucially important in SCA during sports, as they are in other settings. Optimal use of both will most likely result in survival.

- Most videos showing recent events did not show an improvement in the proportion of athletes who received early resuscitation, suggesting that the problem of cardiac arrest during sports activity is poorly recognized.

## Background

Sudden cardiac arrest (SCA) during sport in otherwise healthy athletes is a rare and unexpected event with disastrous consequences, including sudden cardiac death (SCD). Athletes, the role models for our society, are constantly in the spotlight of the media, and unexpected events are extensively discussed through the media. The published media reports typically relate to pre-competition screening and cardiopulmonary resuscitation (CPR).

The annual incidence of SCA/SCD among athletes aged 35 years and younger is 2.2–9.8/100,000 and is consistently lower in females (ratio up to 1:9) and non-athletes (0.31/100,000) [1–5]. It is well-known that physical activity can trigger life-threatening ventricular tachycardia and fibrillation (VT/VF) in (silent) underlying cardiovascular conditions, such as cardiomyopathy, ion channelopathy, and coronary artery disease [4–9]. To prevent SCA/SCD, the international sports governing bodies International Olympic Committee (IOC) and Fédération Internationale de Football Association (FIFA) recommend pre-competition screening of athletes to detect these high-risk cardiovascular conditions (HRCC) [10–13]. However, if SCA occurs despite preventive programs, restoring the circulation first is extremely important to improve survival [4–9, 14–17]. Therefore, the international medical societies and sports associations have provided detailed medical action plans and training programs for handling SCA at sports facilities, including rapid bystander CPR to provide chest compression and defibrillation shocks within 3–5 min using an automatic external defibrillator (AED) on-site [11, 14, 15, 18–24]. However, unexpected episodes of SCA during sport still occur, with uncertain outcomes [11, 18, 19, 25, 26]. To date, four studies reported SCA during sport from video analysis [17, 27–29]. Two studies analyzed the rapidity of bystander CPR in sudden collapse, traumatic or non-traumatic SCA, two studies the circumstances of collapse, and one the features of early recognition of SCA [17, 27–29]. However, external factors causing SCA, such as bodily collision, or situations that can mimic SCA, such as vasovagal collapse, may not refer to underlying HRCC prone for VT/VF, inducing selection bias. Therefore, in this study, we set out to analyze the rapidity of bystander CPR to non-traumatic SCA and outcomes, from video analysis.

## Methods

### Study Design

We searched on images.google.com, video.google.com, and YouTube.com for available videos using the keywords “sudden cardiac arrest athlete,” “sudden cardiac death athlete,” and “resuscitation athlete.” We included any camera-witnessed non-traumatic SCA that occurred in athletes and other sports participants during or shortly after sports participation at any sports facility, by any age, gender, type, and level of sport, that occurred after 1990. Exclusion criteria were traumatic SCA (bodily collision, accident), spontaneous recovery from collapse < 2 min, implantable cardioverter defibrillator (ICD) carrier, and videos inappropriate for assessment.

Next, we searched on Google.com using each victim’s personal name, “cause of cardiac arrest” or “cause of death” as keywords to determine the cause of SCA and survival. This included news reports posted on the internet. We did not review medical records of the victim involved. All data of each victim were anonymized for analysis.

Four observers (NB, PD, LH, MM) collected the obtained videos. The observers were grouped into two pairs to evaluate the included videos. A fifth independent observer (NP) analyzed all obtained and included videos, blinded to the results of the two pairs of observers. Disagreement between the two pairs and the fifth observer was resolved by consensus.

We determined each victim’s baseline characteristics, such as age, sex, ethnicity, sports discipline, level of sport, year, and country of event. To determine recognition of SCA from the videos, we evaluated for each victim the physical activity immediately before onset of SCA, the mode of collapse during SCA onset, the appearance during on-going SCA (body position, movements, facial expression), and the nature and rapidity of bystander CPR to SCA. We did not assess agonal respirations or gasping.

To determine the main outcome of our study, the rapidity of bystander CPR, we assessed the time from SCA onset to starting chest compressions and from SCA onset to defibrillation, using a stopwatch. Time was measured in minutes and seconds. Considering that the ideal time from sudden collapse to receiving a defibrillation shock (if required) should be within 3–5 min, we classified the rapidity of bystander CPR as < 3, 3–5, or > 5 min. If the video recording was not displayed continuously beyond 5 min and chest compressions nor defibrillation were initiated within 5 min, we assessed the time as > 5 min.

### Definitions

We defined “athlete” as an individual who participates in an organized team or individual sport competing against others on a regular basis aiming to improve skills, excellence, and athletic achievements. This includes high-school and collegiate sport. “Elite athlete” is an athlete who competes at the highest level of national and international competition. “Recreational sports participant” is an individual performing leisure-time activity. “Victim” is any athlete, elite-athlete, or recreational sports participant in SCA.

### Statistical Analysis

In this descriptive observational study, we presented continuous variables with means and standard deviation (SD) and median values and interquartile range (IQR) for non-normal distributed variables. We presented categorical variables as the number of patients and percentages. We conducted statistical analysis using the SPSS package version 26.0 (SPSS® Inc., Chicago, IL, USA).

## Results

**Fig. 1:**
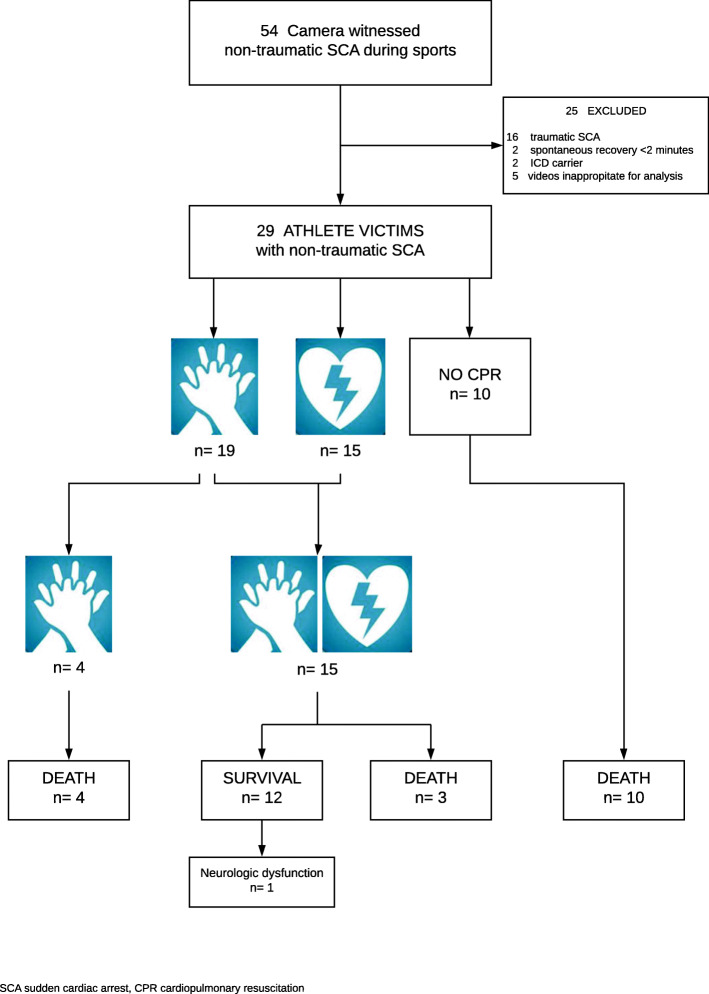
Utstein-style camera-witnessed non-traumatic sudden cardiac arrest in athletes summary. Legend: SCA, sudden cardiac arrest; CPR, cardiopulmonary resuscitation

In total, we identified and included 29 victims with camera-witnessed non-traumatic SCA during sports in our analysis (Fig. 1). Individual summaries of each victim are shown in Table 1.

**Table 1:** Summary of camera witnessed non-traumatic sudden cardiac arrest in 29 victims during sports

Victim No.	Baseline characteristic (year of event)	Bystander response	Time to start chest compressions and defibrillation (min)	Survival, cause of SCA (intervention)
1	Male, 33y., BA	opening airway, CC: no	-	died, HCM
	basketball, elite (1990)	AED: no	-	
2	Male, 28y., BA	CC: no	-	died, HCM
	soccer, elite (2003)	AED: no	-	
3	Male, 25y., WA	CC: physician	CC: 2.36	died, HCM
	soccer, elite (2004)	AED: physician	AED: 5.15	
4	Male, 30y., Hispanic	CC: yes	CC: 0.31	died, HCM
	soccer, elite (2004)	AED: no	-	
5	Male, 21y., WA	CC: paramedic/physician	CC: >5	survived, AVC (ICD)
	soccer, elite (2005)	AED: yes	AED: >5	
6	Male, 35y., WA	CC: yes	CC: >5	died, ion channelopathy
	soccer, elite (2007)	AED: yes	AED: >5	
7	Male, 23y., WA	opening airway, CC:no	-	died
	soccer, elite (2008)	AED: no	-	
8	Male, 23y., BA	CC: paramedic/physician	CC: 1.50	survived, HCM (ICD)
	soccer, elite (2010)	AED: physician	AED: 1.50	
9	Male, 31y., WA	CC: yes	CC: 0.54	survived, ACS
	soccer, elite (2010)	AED: yes	AED: >5	
10	Male, 23y., BA	CC: no	-	died
	soccer, elite (2010)	AED: no	-	
11	Male, 31y., Hispanic	CC: yes	CC: 1.07	survived
	soccer, elite (2010)	AED: paramedic	AED: 1.50	
12	Male, 24y., BA	CC: yes	CC: 1.07	survived, HCM (ICD)
	soccer, elite (2012)	AED: yes	AED: >5	
13	Male, 25y., WA	CC: physician	CC: 2.50	died, AVC
	soccer, elite (2012)	AED: no	-	
14	Male, 52y., WA	CC: teammate	CC: 3.55	survived
	basketball, competition (2013)	AED: teammate	AED: 1.50#	
15	Male, 27y., WA	opening airway, CC: no	-	died, ion channelopathy
	soccer, elite (2015)	AED: no	-	
16	Male, 19y., BA	opening airway, CC: no	-	died, HCM
	soccer, elite (2016)	AED: no	-	
17	Female, 17y., WA	CC: parents/other	CC: 0.48	survived, ACA (CABG & ICD)
	volleyball, competition (2016)	AED: parent/other	AED: 2.50	
18	Male, 26y., BA	CC: paramedic	CC: 2.25	died
	soccer, elite (2016)	AED: no	-	
19	Male, 20y., BA	CC: paramedic	CC: 12.56	survived with severe neurologic dysfunction, iVT
	soccer, elite (2017)	AED: paramedic	AED: 13.10	
20	Male, 39y., BA	CC: no	-	died
	volleyball, elite (2017)	AED: no	-	
21	Male, 16y., WA	opening airway, CC: no	-	died
	volleyball, high school (2017)	AED: no	-	
22	Male, 15y., BA	CC: teammates	CC: 1.50	survived
	basketball, competition (2017)	AED: teammate	AED: 2.50	
23	Male, 49y., WA	CC: sparring partner	CC: 0.20	survived, ACS (PCI & stent)
	karate, competition (2017)	AED: paramedic	AED: 2.20	
24	Male, 23y., other race	opening airway, CC: no	-	died
	cricket, elite (2018)	AED: no	-	
25	Male, 25y., WA	CC: physician	CC: 1.50	died
	soccer, elite (2018)	AED: no	-	
26	Male, 23y., WA	CC: paramedic	CC: >5	died, no structural HD
	cycling, elite (2018)	AED: paramedic	AED: >5	
27	Male, 26y., BA	CC: no	-	died
	basketball, elite (2018)	AED: no	-	
28	Male, 52y., WA	CC: physician	CC: 0.20	survived, ACS (PCI & stent)
	icehockey, recreational (2019)	AED: physician	AED: 0.35	
29	Male, 29y., WA	CC: paramedic	CC: 1.57	survived, (ICD)
	soccer, elite (2021)	AED: paramedic	AED: 2.50	

Of the victims, 28/29 were males (96.5%), 15/29 white athletes (51.7%), 23/29 elite athletes (79.3%), and 18/29 participated in soccer (62.1%). The average age was 27.6 ± 8.5 years (range 15–52) years. Videos of 14/29 victims (48.6%) were recorded in Europe.

**Fig. 2:**
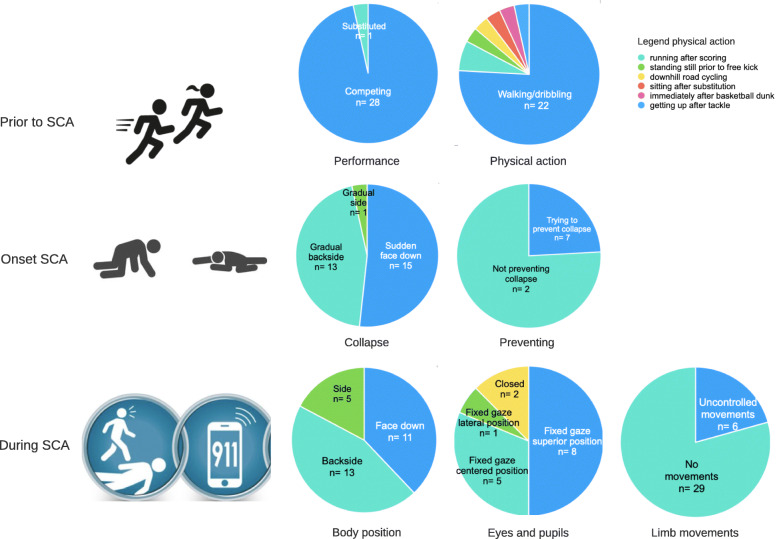
Athlete’s behavior prior to and during sudden cardiac arrest. Legend: SCA, sudden cardiac arrest

Before SCA occurred, 28/29 victims were competing (96.5%), 22/29 performed low-intensity exercise (75.9%) (Fig. 2). During the onset of SCA, 13/29 victims gradually collapsed on their back (44.8%), 15/29 suddenly dropped face down (51.7%), and 7/29 tried to prevent collapse (24.1%). During SCA, the most common feature was fixed gaze in 18/18 victims with visible and open eyes. Uncontrolled limb movements were observed in 6/29 (21.4%) (Fig. 2).

**Fig. 3:**
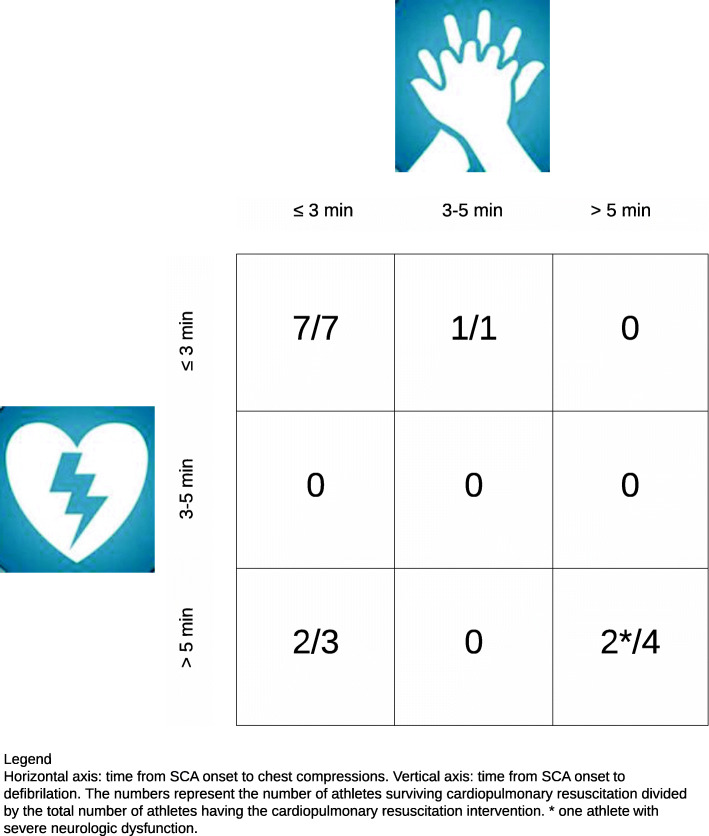
Athletes surviving cardiopulmonary resuscitation. Legend: Horizontal axis, time from SCA onset to chest compressions. Vertical axis, time from SCA onset to defibrillation. The numbers represent the number of athletes surviving cardiopulmonary resuscitation divided by the total number of athletes having the cardiopulmonary resuscitation. * one athlete with severe neurologic dysfunction

Of all victims analyzed, 12/29 survived (41.4%) (Fig. 1). Bystanders performed chest compression and defibrillation in 15/29 victims (51.7%), and chest compressions alone in 4/29 (13.8%) (Fig. 1). The latter four died. Ten victims received neither chest compressions nor defibrillation (34.5%). All 10 died. In 6/29 victims, bystanders concentrated on trying to open the airway without giving chest compressions (20.7%). Medical and paramedical personnel performed chest compressions in 10/29 victims (34.5%) and defibrillation in 8/29 (27.6%). Referees did not perform chest compressions in any of the videos analyzed. Eight victims received defibrillation < 3 min, in seven of them chest compressions were < 3 min, and in one 3–5 min (median time to start chest compressions 1.50 min, median time to defibrillation 2.50 min) (Fig. 3). The latter received defibrillation before compressions. All eight survived. Delaying both chest compressions and defibrillation beyond 5 min was associated with worse outcome: death 4/29 (13.8%) and neurologic dysfunction 1/29 (3.4%). We could not calculate the IQR, because we indicated the rapidity as > 5 min for chest compressions in three victims and for defibrillation in five.

The cause of SCA was reported in 17/29 victims. One was reported as “no structural heart disease,” 16 had HRCC, hypertrophic cardiomyopathy (7), arrhythmogenic cardiomyopathy (2), ion channelopathy (2), idiopathic VT (1), anomalous coronary artery origin (1), coronary artery occlusion (3)

## Discussion

Our analysis of the internet videos of 29 victims showed that immediate bystander CPR to non-traumatic SCA during sport was associated with improved survival. This suggests that immediate chest compressions and defibrillation within 3 min are crucially important in SCA and will most likely result in survival. Almost all SCA victims caught on video were elite athletes, with an organized medical team and emergency equipment including AED for immediate chest compressions and defibrillation on-site. However, videos showing recent events did not show an improvement in the proportion of athletes who received early resuscitation, suggesting that the problem of SCA during sports activity is poorly recognized. Seventy-six percent performed low-intensity exercise before SCA occurred. During the onset of SCA, 51.7% collapsed suddenly face down and 48.3% gradually on their back or lateral side. The most common feature during SCA was a fixed gaze.

The first important question is why to date athletes still die at the sports facility? SCA may occur in every individual and is the leading cause of death in sport [11]. Survival from SCA is determined by early recognition, immediate bystander chest compressions, and early defibrillation without any hesitation. In this study, it appears that bystanders do not always recognize SCA. Viskin et al. reported in a similar camera-witnessed study of 23 traumatic or non-traumatic SCA and six sudden collapses in athletes during sport that bystanders failed to recognize SCA and tried to open the airway without performing chest compressions in 72.4% [27]. In our study, this was 21.4%. Contrary to Viskin et al., we included non-traumatic SCA only to exclude external factors causing SCA, such as blunt chest trauma that may induce asystole or VT/VF depending on the timing of the cardiac cycle the impact occurred [30]. In a previous study, we used the same study method in six elite athletes suffering non-traumatic SCA during sport [17]. We observed in all six victims during the initiation of syncope an unexpected sudden loss of the upright position, loss of normal breathing, and a fixed gaze [17]. In this study of 29 victims, we found the fixed gaze in all if the eyes were visible. Recognition of the lethal situation of SCA is an important topic in CPR and AED training for medical and paramedical personnel, team staff, and referees [11, 19, 24]. Although referees are included in CPR training programs, we did not observe referees, who were nearby to a victim (especially in soccer), performing chest compressions. In soccer, FIFA uses their standard protocol field-of-play signs of SCA and organizes hands-on and electronic CPR courses regularly to FIFA venue medical officers, football team physicians, and physiotherapists [24]. Maybe, referees can attend these courses too? Nevertheless, if an athlete suddenly collapses during sport for no apparent reason and is unresponsive and not breathing normally, bystanders should perform CPR immediately without any hesitation or delay in starting chest compressions [21]. One bystander should start chest compressions, whilst others should call the local emergency medical services (EMS) (911 US, 112 EU) and fetch an AED and apply it. Bystanders should follow the instructions given by the AED concerning chest compressions, analysis of the cardiac rhythm, and defibrillation [15].

The remaining question is why there was such a defibrillation delay of beyond 5 min? It is widely accepted that defibrillation within 3–5 min increases survival (50–70%) [15]. AEDs can be life-saving as is demonstrated by a Swedish (*n* = 474) and a Dutch study (*n* = 320) [31, 32]. Both studies observed that survival from out-of-hospital cardiac arrest—if AED was applied—was 70% and 52%, respectively [31, 32]. However, if bystanders performing CPR were interrupted by EMS paramedics or the application of the next AED shock was delayed, survival was reduced [32]. Drezner et al. demonstrated in a 2-year prospective study in 2149 American high schools equipped with an AED, that on-site defibrillation within 3.5 min was associated with a survival of 89% (42/59 victims) [6]. Of the included 59 SCA events, 54 were witnessed, AED was applied in 50 victims, and 39 of them received defibrillation [6]. Bohm et al. reported from a prospective German registry of SCA during sport (*n* = 144) a survival rate of 26.4% [9]. The authors found that immediate bystander CPR was performed in 82%, and 40.7% had a shockable rhythm [9]. The survival rate in the German study among predominantly male middle-aged sports participants was lower than in the Swedish and Dutch studies among the general population [9, 31, 32]. However, our observations of a relatively small and selected cohort should raise concern with guideline committees and CPR trainers for EMS paramedics, physicians, and other personnel witnessing athletic activities in being trained for basic life support (BLS) and AED [14–16, 18]. The time from SCA onset to defibrillation is the crucial determinant of survival. In a French registry of exercise-related SCA (*n* = 820), the authors observed delay of AED arrival (beyond 6 min) to indoor- and outdoor-sports facilities, and infrequent use of an AED (< 1%) [7]. They reported higher survival in indoor-sports facilities (23%) than in outdoor-sports facilities (8%) [7]. In addition, it was noted that not performing or delaying AED shocks explained low survival. Furthermore, public access availability of AEDs and implementation of AEDs in high schools emergency action programs were more likely to early defibrillation [33–35]. AEDs should also be accessible at sports clubs/facilities. Steinkog et al. found in a similar study reviewing 26/35 videos of traumatic and non-traumatic SCA and arrhythmia-collapse in athletes, an association between very rapid bystander CPR and high survival (100%) [28]. Survival was 92% if defibrillation was ≤ 1 min (*n* = 12) [28]. In our study with different inclusion criteria, we found an important association between immediate chest compressions and defibrillation within 3 min and 100% survival in eight victims (27.6%). As described by Steinskog et al. and Zorzi et al., we also observed most athletes performing low-intensity exercise before SCA occurred, the analysis of which was beyond the scope of our study [28, 29].

Athletic activities are often witnessed by many spectators, and therefore if SCA occurs there are more bystanders present to respond to SCA. However, it is difficult to explain that sometimes bystanders hesitate or delay immediate chest compressions and defibrillation, and that other bystanders do not take their responsibility to start chest compressions. AEDs are recommended to be available on-site during athletic events and laymen are allowed to use them [14, 18, 19, 21]. In the FIFA 11 steps to prevent SCD in soccer, FIFA recommends to put a medical emergency bag with an AED in position besides the field-of-play and to check it before each professional soccer match [11]. In addition, if SCA occurs, the AED should be retrieved, applied, and used as soon as possible [11]. Unfortunately, even to date, a rapid emergency action sometimes fails. Fortington et al. reported in an Australian survey, an increasing number of AED trained individuals at sports clubs/facilities following a government-led program [36]. However, 33% of the 191 respondents did not know whether their sports club/facility had an emergency action plan, meaning room for improvement. The authors concluded that the AED can only be effective when people are confident with the CPR process and know where to locate the AED [36].

Other avoidable (or unavoidable) delays are indecisiveness during resuscitation or emotional blockade of the bystander to start CPR. Groombridge et al. found in a systematic review heterogenous human stressors that can hinder decision-making in CPR, among which illness severity, victim deterioration, stress from uncertainty, and time pressure during CPR [37]. Wik et al. analyzed the quality of CPR in 176 out-of-hospital advanced cardiac life supports procedures in the general population [31]. The authors found that bystanders, highly trained paramedics, and anesthetists failed to perform chest compressions in 48% of cases, probably because of an emotional blockade to perform CPR [38]. Hasselqvist-Ax et al. analyzed bystanders’ experience among 10 police officers and 12 fire fighters [39]. Lack of information about the victim during and after CPR, uncertainty about their skills to perform CPR, and psychological reactions of bystanders led to emotional stress [39]. Zijlstra et al. conducted a prospective observational study by e-mail questioning emotional distress within 4–6 weeks after CPR attempt [40]. Among 1955 first responders and 507 assisting CPR, 13% experienced severe psychological impact, but none of the participants experienced post-traumatic stress [40]. Not connecting and AED to the victim was one of the stress causing factors [40]. In our study, many bystanders were on the professional athletic team and therefore had a more personal relationship with the victim. Lack of personal information could not have been the problem, but lack of the clinical information, victim deterioration, and/or emotional blockade could have been stressors hindering the CPR procedure. Furthermore, it seemed that medical professionals performing CPR at sports facilities did not follow the BLS/AED algorithm, which also implies substantial room for improvement [15, 16, 34]. Techniques to overcome the emotional stress were beyond the scope of our study. However, Groomberg et al. found in the systematic review mentioned above that tailored stress training, simulation of cognitive aids, checklist for the management, and mindfulness meditation may overcome stressors [37].

In our study, most athletes participated in commonly filmed elite sports, such as soccer. However, it does not reflect the risk of SCA in soccer compared to other sports. We found no camera-

witnessed SCA in other popular sports, such as marathon running. During mass events with thousands of participants, the chance of capturing SCA on video is extremely small. Nevertheless, it is unacceptable that athletes still die during sports and that despite BLS/AED training and the availability of AED on-site, bystander CPR to SCA is delayed or absent. Our data strongly suggest that more SCA victims survive if bystander CPR is rapid and any delay in starting chest compressions and defibrillation is avoided.

## Strengths and Limitations

The main strength of our study is highlighting once again the importance of increasing the number of CPR trained lay bystanders and number of available AEDs in sports facilities and competitions. Still too often and even in elite/professional sports, we see inadequate responses to SCA events.

Our collection of victims was limited to the included videos that were posted by television stations, athletic organizations, and individuals recording SCA and CPR. Therefore, the information is selected and heterogeneous, introducing selection and performance bias. Many uploaded videos were from European countries showing popular elite male sporting events. Non-European countries, females, less popular sports, and other levels of sport were underrepresented, drawing solid conclusions uncertain. Nevertheless, our information is relevant and valuable. The main advantage of the camera-witnessed analysis was to objectively review victims’ behavior before, at the onset and during SCA, and most importantly the rapidity of bystander CPR to SCA. We did not assess the quality of bystander CPR. The technical aspects of CPR, such as the depth and rate of chest compressions, defibrillation, and ventilation were beyond the scope of our study. In some victims, the exact time to chest compressions and defibrillation could not be measured and was set at > 5 min. Survival improved if chest compressions were performed within 5 min. Therefore, we included these > 5 min results in our analysis.

We excluded traumatic SCA including bodily collision from our study. However, it could be possible that we may have missed commotio cordis as a cause of SCA.

Our cohort is not a representative sample of SCA occurring during sport. Most included victims were male elite athletes competing in popular sport like soccer, especially in Europe. Notwithstanding these limitations and the small numbers, our study suggests that it is a continuous challenge to improve the recognition of SCA and bystander CPR to SCA during sport.

## Future Directions

To improve early recognition of SCA immediately followed by chest compressions and defibrillation without any hesitation, we propose that everyone involved in sports events is encouraged to attend BLS/AED training, thereby increasing SCA awareness and BLS/AED familiarity. This applies to teammates, coaching staff, referees and jury members, and especially supporting medical and paramedical professionals. Referees and jury members are part of the competition and are nearest to a potential victim.

As suggested by experts’ opinions, every sports club/facility/organization and school should have a written emergency action plan, AED accessible on-site, and organize CPR training preferably starting at school-age (from age 10 to 12 years). Finally, as suggested in the literature, CPR training programs should also address the mental status of bystanders, including medical and paramedical professionals, to ensure adequate emergency action during the stressful circumstances of SCA. This could be achieved for instance, by organizing sports-specific CPR training of the team of athlete(s), coach(es), medic(s) and paramedic(s), and eventually officials. During the CPR training, the process of effectively working together as a resuscitation team can be trained and “human factors” can be eliminated, resulting in prompt and appropriate bystander CPR if unexpected SCA during sport occurs.

## Conclusions

Analysis of internet videos showed that immediate bystander CPR to non-traumatic SCA during sport was associated with improved survival. This suggests that immediate chest compressions and early defibrillation are crucially important in SCA during sport, as they are in other settings. Optimal use of both will most likely result in survival. Videos showing recent CPR suggest that the fatal situation of SCA is poorly recognized.

## Data Availability

In the “Methods” section, we mentioned that “All victim’s data were anonymized for analysis” to protect the victim’s privacy. We mentioned in the legend of Table 1 “Note: References of the videos/images are not displayed due to privacy regulations.” As we do not intend to violate the privacy regulations, we decided not to mention the references of the internet sites displaying the videos. The datasets used and analyzed during the current study are available from the corresponding author on reasonable request.

## Abbreviations

AED: Automatic external defibrillator
BLS: Basic life support
CPR: Cardiopulmonary resuscitation
EMS: Emergency medical services
EU: Europe
FIFA: Fédération Internationale de Football Association
HRCC: High-risk cardiovascular conditions
ICD: Implantable cardioverter defibrillator
IOC: International Olympic Committee
IQR: Interquartile ranges
SCA: Sudden cardiac arrest
SCD: Sudden cardiac death
SD: Standard deviation
VF: Ventricular fibrillation
VT: Ventricular tachycardia

## References

[1] Corrado D, Basso C, Rizoli G (2003). Does sports activity enhance the risk of sudden death in adolescent athletes and young adults?. J Am Coll Cardiol.

[2] Marijon E, Tafflet M, Celermajer DS (2011). Sports-related sudden death in the general population. Circulation.

[3] Thoresdahl BG, Al R, Harmon KG (2014). Incidence of sudden cardiac arrest in high school student athletes on school campus. Heart Rhythm.

[4] Corrado D, Basso C, Pavei A (2006). Trends in sudden cardiovascular death in young competitive athletes after implementation of a pre-participation screening program. J Am Medical Assoc.

[5] Harmon KG, Asif IM, Maleszewski JJ (2015). Incidence, etiology, and comparative frequency of sudden cardiac death in NCAA athletes: a decade in review. Circulation.

[6] Drezner JA, Toresdahl BG, Rao AL (2013). Outcomes from sudden cardiac arrest in US high schools: a 2-year prospective study from the National Registry for AED Use in Sports. Br J Sports Med.

[7] Marijon E, Bougouin W, Karam N (2015). Survival from sports-related sudden cardiac arrest: In sports facilities versus outside of sports facilities. Am Heart J.

[8] Solberg EE, Borjesson M, Sharma S (2016). Sudden cardiac arrest in sports - need for uniform registration: a position paper from the Sports Cardiology Section of the European Association for Cardiovascular Prevention and Rehabilitation. Eur J Prev Cardiol.

[9] Bohm P, Scharhag J, Meyer T (2016). Data from a nationwide registry on sports-related sudden cardiac death in Germany. Eur J Prev Cardiol.

[10] Corrado D, Pelliccia A, Bjornstad HH (2005). Cardiac pre-participation screening of young competitive athletes for prevention of sudden death: proposal for a common European protocol. Consensus Statement of the Study Group of Sport Cardiology of the Working Group of Cardiac Rehabilitation and Exercise Physiology and the Working Group of Myocardial and Pericardial Diseases of the European Society of Cardiology. Eur Heart J.

[11] Dvorak J, Kramer EB, Schmied CM (2013). The FIFA medical emergency bag and FIFA 11 steps to prevent sudden cardiac death: setting a global standard and promoting consistent football field emergency care. Br J Sports Med.

[12] Maron BJ, Levine BD, Washington RL (2015). Eligibility and disqualification recommendations for competitive athletes with cardiac abnormalities: task force 2: pre-participation screening for cardiac disease in competitive athletes. Circulation.

[13] Drezner JA, O'Connor FG, Harmon KG (2016). AMSSM Position statement on cardiac preparticipation screening in athletes: current evidence, knowledge gaps, recommendations and future directions. Br J Sports Med.

[14] Nolan JP, Soar J, Zideman DA (2010). European Resuscitation Council Guidelines for Resuscitation 2010 Section 1.Executive summary. Resuscitation.

[15] Perkins GD, Handley AJ, Koster RW (2015). European resuscitation council guidelines for resuscitation 2015. Section 2. Adult basic life support and automated external defibrillation. Resuscitation.

[16] Berg RA, Hemphill R, Abella BS (2010). 2010 American Heart Association guidelines for cardiopulmonary resuscitation and emergency cardiovascular care. Circulation.

[17] Panhuyzen-Goedkoop NM, Wellens HJ, Piek JJ (2018). Early recognition of sudden cardiac arrest in athletes during sports activity. Neth Heart J.

[18] Link MS, Myerburg RJ, Estes NA (2015). Eligibility and disqualification recommendations for competitive athletes with cardiovascular abnormalities: Task Force 12: emergency action plans, resuscitation, cardiopulmonary resuscitation, and automated external defibrillators: a scientific statement from the American Heart Association and American College of Cardiology. J Am Coll Cardiol.

[19] Borjesson M, Serratosa L, Carre F (2011). Consensus document regarding cardiovascular safety at sports arenas: position stand from the European Association of Cardiovascular Prevention and Rehabilitation (EACPR), section of Sports Cardiology. Eur Heart J.

[20] Siebert DM, Drezner JA (2018). Sudden cardiac arrest on the field of play: Turning tragedy into a survivable event. Neth Heart J.

[21] Hainline B, Drezner JA, Baggish A (2016). Interassociation consensus statement on cardiovascular care of college student-athletes. J Athl Train.

[22] Public Access Defibrillation Guidelines. Federal Register 2001;66:28495-511. The Federal Register Online via GPO Access wais.access.gpo.gov. Accessed 17 Apr 2020.

[23] Kramer E, Bohta M. Emergency cardiac care in the athletic setting: from school sports to the Olympic arena. In: Wilson MG, Drezner JA, Sharma S, editors. IOC manual of sports cardiology, first edition, International Olympic Committee: Wiley; 2017.

[24] Kramer EB, Dvorak J, Schmied C, Meyer T (2015). F-MARC: promoting the prevention and management of sudden cardiac arrest in football. Br J Sports Med.

[25] en.wikipedia.org/wiki/Sudden_cardiac_death_of_athletes. Wikipedia. 2019. Accessed 1 Aug 2019.

[26] en.wikipedia.org/wiki/List_of_association_footballers_who_died_while_playing. Wikipedia, 2019. Accessed 31 July 2019.

[27] Viskin D, Rosso R, Havakuk O (2017). Attempts to prevent “tongue swallowing” may well be the main obstacle for successful bystander resuscitation of athletes with cardiac arrest. Heart Rhythm.

[28] Steinskog DM, Solberg EE (2019). Sudden cardiac arrest in sports: a video analysis. Br J Sports Med.

[29] Zorzi A, Cipriani A, Corrado D (2018). Circumstances of cardiac arrest during sports activity recorded on video. Research letter. Eur J Preven Cardiol.

[30] Link MS (2003). Mechanically induced sudden death in chest wall impact (commotio cordis). Progress Biophysics Mol Biol.

[31] Ringh M, Jonsson M, Nordberg P (2015). Survival after public access defibrillation in Stockholm, Sweden - A striking success. Resuscitation.

[32] Berdowski J, de Beus MF, Blom M (2013). Exercise-related out-of-hospital cardiac arrest in the general population: incidence and prognosis. Eur Heart J.

[33] Blom MT, Beesems SG, Homma PC (2014). Improved survival after out-of-hospital cardiac arrest and use of automated external defibrillators. Circulation..

[34] Zijlstra JA, Radstok A, Pijls R (2016). Overleven na een reanimatie buiten het ziekenhuis: vergelijking van de resultaten van 6 verschillende Nederlandse regio's. Reanimatie in Nederland, 2016.

[35] Toresdahl BG, Harmon KG, Drezner JA (2013). High school automated external defibrillator programs as markers of emergency preparedness for sudden cardiac arrest. J Athletic Train.

[36] Fortington LV, West L, Morgan D, Finch CF (2019). Implementing automated external defibrillators into community sports clubs/facilities: a cross-sectional survey of community club member preparedness for medical emergencies. BMJ Open Sport Exercise Medic.

[37] Groombridge CJ, Kim Y, Maini A (2019). Stress and decision-making in resucitation: a systematic review. Resuscitation.

[38] Wik L, Kramer-Johansen J, Myklebust H (2005). Quality of cardiopulmonary resuscitation during out-of-hospital cardiac arrest. J Am Med Assoc.

[39] Hasselqvist-Ax I, Nordberg P, Svensson L (2019). Experiences among firefighters and police officers of responding to out-of-hospital cardiac arrest in dual dispatch programme in Sweden: an interveiw study. Br Med J Open.

[40] Zijlstra JA, Beesems S, De Haan RJ (2015). Psychological impact on dispatched local lay rescuers performing bystander cardiopulmonary resuscitation. Resuscitation.

